# Lived experience perspectives on labeling and defining long-standing anorexia nervosa

**DOI:** 10.1186/s40337-021-00457-x

**Published:** 2021-08-14

**Authors:** Catherine Broomfield, Paul Rhodes, Stephen Touyz

**Affiliations:** 1 School of Psychology, Griffith Taylor Building, The University of Sydney, Sydney, NSW 2006 Australia; 2 InsideOut Institute, Charles Perkins Centre, The University of Sydney, Sydney, NSW 2006 Australia

**Keywords:** Anorexia nervosa, Long-standing, Severe and enduring, Chronic, Labeling, Defining

## Abstract

**Objective:**

Since efforts to stage anorexia nervosa (AN) revealed the existence of various presentations, research into the long-standing subgroup has increased. A change in treatment has been proposed with the intention to use more effective evidence-based methods that target symptoms of the long-standing presentation and improve prognosis. A barrier in achieving this goal in both research and clinical contexts is the lack of a consistent label and definition. This makes the ability to assess, recruit and treat these presentations difficult. Investigations into how this subgroup may be differentiated from other stages of the disorder have included the opinions of practitioners and researchers with little consideration for the perspectives of individuals living with this illness. It was the aim of the current study to investigate lived experience perspectives on the way long-standing AN should be labeled and defined.

**Methods:**

Data were collected through a semi-structured interview within a narrative inquiry framework. This approach is beneficial when examining processes that occur over time, such as investigations into a long-term illness. A total of 11 women with a presentation of long-standing AN participated in an interview. Data were divided into two categories for analysis based on the association to labeling or defining the features of the long-standing AN illness.

**Results:**

Two labels emerged during the analysis with participants describing a preference for the use of ‘severe and enduring’ over ‘chronic’ when referring to their presentation of AN. When defining the illness, the most preferred criterion was illness duration with mixed perspectives for the use of previously failed treatment attempts. Participants described a consistent dislike for the use of low body weight as a feature in the defining of the illness.

**Conclusions:**

The current study describes how individuals with a lived experience prefer to have the long-standing AN presentation labeled and defined. It is the hope of the authors that these insights will be adopted into any guidelines developed to ensure individuals most affected by this disorder have a voice and continue to be given the opportunity to contribute to topics related to their illness.

**Plain English summary:**

Anorexia nervosa (AN) is a complex illness that has been divided into stages based on the severity of symptoms. Little is known about the AN stage that persists over lengthy periods of time with research pursuits underway to determine characteristics that allow this disorder to persevere. A barrier in researching and treating these individuals is the lack of a consistent label to refer to these presentations and criteria that will allow us to identify this stage of AN. The aim of the current study was to determine how individuals with a lived experience of long-standing AN prefer to have their illness labeled and defined. A total of 11 women who had experienced this stage of AN were interviewed with the majority of participants reporting to prefer the label ‘severe and enduring’ over the term ‘chronic’. Additionally, most of the participants had a preference for defining their illness based on the duration of time the illness had persisted with mixed opinions for using the number of previously unsuccessful treatment attempts as criterion. The authors are hopeful that any guidelines established for labeling and defining long-standing AN will incorporate the perspectives of individuals with a lived experience of the illness.

## Background

Anorexia nervosa (AN) is a complex psychiatric illness that manifests physically and psychologically [1]. Symptoms of the illness include low body weight, disordered eating behaviours, an intense fear of becoming fat and a disturbance in the way the individual perceives their own body shape [2]. With variability in its presentation across individuals, efforts have been made to stage the illness based on the premise that symptoms of AN exist along a continuum of severity [3–5]. Following this line of research, the pursuit into understanding and treating the later stage of AN has been undertaken given individuals that present with this form of the disorder currently experience a poor prognosis (see [6] for a review).

One of the ways in which both research and clinical practice has been hindered is through the difficulty in identifying presentations of AN, which have progressed into the later stage of the illness. With no single universal definition or guidelines currently available [7–9], practitioners have been forced to use their own criteria when recruiting individuals for research. A further complication is the inconsistent labeling of this subgroup of patients, with various terms currently being used including ‘severe and enduring’ AN (SE-AN) and ‘chronic’ AN [7]. This precludes the ability of researchers and clinicians to remain updated on progress with respect to the understanding of characteristics relating to these unique presentations. With an estimated 20% of individuals experiencing a long-term trajectory of the illness [10], it is imperative that a universal label and definition be actively pursued so that the field can unite in working towards improving the outcome for these patients.

In a study by Tierney and Fox [11], a Delphi method was utilised in order to reach consensus on a definition of chronic AN by a sample of practitioners. It was determined that entrenched patterns of food restriction, entrenched ‘anorexic’ cognitions, an identity intertwined with AN and a low body mass index (BMI) were criteria that were agreed upon by eating disorder professionals as constituting a chronic presentation of AN. However, a consensus on two additional criteria were unable to be reached; that is, duration of illness and the number of unsuccessful treatment attempts that needed to be made by patients [11].

Despite these findings, one of the more popular trends in the field of eating disorders has been to recruit participants based on illness duration as the key criterion. With only one randomised controlled trial conducted on the psychological treatment of SE-AN to date, Touyz et al. [12] used an illness duration of seven or more years of AN as an inclusion criterion for participation. Following this study, a high volume of research on SE-AN in the field has utilised this definition through the numerous papers that have stemmed from this primary study [13–16]. Additionally, other research studies have since adopted this definition for the recruitment of participants [17, 18]. A systematic review that was conducted by Broomfield et al. [7] identified the popularity of this criterion in the literature with illness duration (most consistently a seven or more year experience of AN) highlighted as the most common definition in publications in the field, followed by the number of unsuccessful treatment attempts.

Research has since been dedicated to the pursuit of the characteristics of this subgroup. Wildes et al. [19] conducted the first study to use empirical methods in an attempt to develop an evidence-based definition of SE-AN. The authors described that the best-fitting model involved the use of the severity indicators of quality of life and eating disorder behaviours, as well as external factors including age, weight concerns, lifetime mood and anxiety comorbidity. Illness duration and number of unsuccessful hospital admissions were unsupported in differentiating subgroups in their sample [19]. Recent research that was conducted by Ambwani et al. [20] revealed the combination of a psychological distress indicator and duration of illness to be what best differentiated severe and enduring individuals from participants at earlier stages of the disorder. The outcome variable used to determine its efficacy were the symptomatic changes that occurred over 12 months with the participants within the severe and enduring condition demonstrating ongoing impairment in both work and social adjustment, as well as a reduced likelihood to have an increase in body weight [20].

What all of the research to date has in common is that it has implemented the judgment of practitioners and used empirical tools to investigate the optimal way to label and define this form of the illness. What has failed to be considered in research to date, however, is the perspectives of individuals who have a lived experience of the illness. The need to include these alternative points of view in the debate is of paramount importance given these individuals have experienced the effects of this illness. This provides them with a unique perspective that cannot be substituted by clinical experience. Until these individuals are considered and their opinions heard, the field cannot effectively progress.

With underlying processes unable to be captured using the quantitative methods that have so far been employed, the current study adopted a qualitative approach into this topic. It was the aim of the authors to investigate lived experience perspectives on the way in which long-standing AN^1^ should be labeled and defined. It was hoped that by providing these individuals the opportunity to contribute to this debate, the process would empower this often marginalised group [21–23]. It was the goal of the authors that insight provided by individuals with lived experience would be incorporated into guidelines that eventually lead the field of eating disorders forward by providing a universal label and definition for this later stage of the AN illness.

## Method

This study is a secondary analysis of data from a qualitative investigation on long-standing AN, which was conducted between August 2017 and January 2019. A detailed description of the method is reported in the main outcome article [24] with a brief description provided below.

### Participants

Participants were required to meet the following self-reported inclusion criteria: (a) aged at least 18 years; (b) currently or previously met the Diagnostic and Statistical Manual of Mental Disorders, 5th Edition criteria for AN [2]; and (c) had an illness duration of at least seven years. Inclusion criterion (c) was adopted for the current study given it was determined to be the most commonly cited definition in the literature when recruiting participants that are suspected of experiencing the later stage of AN [7]. Snowball sampling was the method of recruitment utilised, which involved seeking the social networks of participants to provide contacts of other potentially interested individuals [25], together with advertising through social media and clinician recommendation in Sydney, Australia. Information on inclusion criteria and details of the study were provided through email following initial contact. Individuals of any gender, ethnic background and socio-economic status that met inclusion criteria and provided written consent were invited to participate. In compliance with the Human Research Ethics Committee at the University of Sydney, all participants provided written informed consent with participants offered no monetary or other forms of compensation.

### Research design

Following the collection of demographic information through a questionnaire, a semi-structured interview was conducted within a narrative inquiry approach. This was deemed an appropriate research framework given narrative inquiry is beneficial when examining processes that occur over long periods of time [26]. With stories shaping identity formation, which is a crucial component for determining the representation of an illness in the lives of individuals, the use of narrative provided a platform for discovering biological and psychological processes, which are embedded within a sociocultural context. These dimensions of an individual were crucial to ascertain in order to gain a comprehensive and meaningful understanding as to the preferences of participants when it comes to labeling and defining their long-standing AN condition [27, 28]. Processes that had occurred throughout the lived experiences of the illness were investigated in each story (as reported by Broomfield et al. [24]), which provided a foundation for understanding the spectrum of opinions described by the sample. Following the telling of each story, a semi-structured interview was conducted in order to gain specific information on preferences relating to the representation of their illness [29].

### Data collection and procedure

Data collection took place through face-to-face interviews or via Skype. The semi-structured interview began with an exploration of the experiences that participants had endured with their long-standing illness from childhood to the date of the interview. Participants were asked to recall details relating to their illness, which was found to vary between experiences. By adopting this epistemological framework, participants were considered the experts on their experience with the researcher discovering new meaning by assimilating the stories told [30]. Critical life events relating to AN emerged, providing rich detail for the focus of the current study and context for what had led individuals to their perspectives on illness representation.

Following a description of lived experience, participants were asked pre-planned questions relating to the topic (see Table 1 for interview questions) together with follow up questions based on the answers provided in order to gain additional detail on illness representation [31]. The entire process took approximately two hours and 30 min, with the semi-structured interview taking 30 min to complete. Interviews were audio-recorded, which were then transcribed verbatim with participants offered the opportunity to member check (process of allowing participants the opportunity to review the analysis and make any changes they request) [32].

**Table 1:** Interview questions

Is there a particular label you prefer for the type of anorexia nervosa that you are experiencing? If so, can you explain to me why?	
How do you think severe and enduring anorexia nervosa should be defined?	

### Data analysis

With different forms of data more suited to certain analytical procedures [28], data relating to the narrative of individuals were analysed for themes using an inductive process, which has been reported in the main outcome article [24]. To address the aims outlined in the current study, a tallying approach was adopted with descriptions provided by participants used to inform and support their interpretation. The use of tallying was similarly applied in research investigating the labeling and defining of SE-AN in the literature by Broomfield et al. [7]. In the current study, transcripts were analysed with comparable descriptions and common terminologies identified [33]. This involved dividing data based on its relevance to the labeling and defining of the illness with each question being analysed separately.

Descriptions relating to the labeling and defining of the illness were categorised based on the likes, dislikes and indifferences of participants. Quotations that reflected the preference of labels and criterion were also extracted from the transcripts in order to preserve the “voice” of participants. After all the data relating to labeling and defining were categorised, the adjectives used to refer to long-standing AN and criteria used to define this stage of the illness were tallied in order to identify the preferences of the lived experience sample. Additional descriptions provided by participants were reported including reasons for liking or disliking certain terminology and criteria that had been used to identify their prolonged experience of AN.

The process of analysis involved data being cross-coded by two authors with any disagreements resolved through discussion. The recruitment of participants ended when inductive saturation was demonstrated, which is the process whereby no additional themes emerge from the data analysis [34].

### Methodological rigor

By adopting a narrative inquiry framework, an understanding of experiences, interests, relationships and positions in life were gained. This strengthened the validity of the findings through an increased awareness as to the network of meanings, which was a more suitable approach to collecting data on the topic of illness representation compared to an investigation in isolation without the context of lived experience [35]. Validity was also strengthened in the current study by offering participants the opportunity to member check [32]. In order to maintain credibility, direct quotes were used from participants together with participant characteristics collected to facilitate an understanding of fittingness. The criterion of auditability was demonstrated through the keeping of a thorough audit trail ensuring analytic transparency [36].

## Results

Out of 26 individuals expressing interest in the study, a total of 11 women participated after self-reporting having met the inclusion criteria. A detailed description of participant demographics (as reported in the main outcome article by Broomfield et al. [24]) is available in Table 2.

**Table 2:** Participant demographics

Characteristic	*n* (proportion)	Mean (SD; years)	Range (years)
Age		41.6 (11.5)	29–66
Duration of illness^a^		26.2 (13.2)	7–53
*Stage of illness*			
Currently ill	6 (54.55%)	–	–
Recovering	3 (27.27%)	–	–
Recovered^b^	2 (18.18%)	–	–
*AN subtype*			
Restricting	9 (81.82%)	–	–
Binge-eating/purging	2 (18.18%)	–	–
*Employment status*			
Unemployed	4 (36.36%)	–	–
Casual	2 (18.18%)	–	–
Part-time	1 (9.09%)	–	–
Full-time	4 (36.36%)	–	–
*Marital status*			
Single	8 (72.73%)	–	–
De-facto/married	3 (27.27%)	–	–
*Children*			
No	8 (72.73%)	–	–
Yes	3 (27.27%)	–	–
*Language spoken at home*			
English	11 (100%)	–	–

AN = Anorexia Nervosa. BMI was not collected as irrelevant for the current study

^a^Each participant provided their own indication of when their illness began. The decision to have women decide the illness starting point was in line with the current research method of working with lived-experience participants who are regarded as the expert on their experience. The majority of participants regarded the starting point of their illness to be when they themselves first noticed symptoms of AN (*n* = 10), with some of these women (*n* = 2) also receiving a diagnosis by a health professional the same year symptoms began. The remaining participant (*n* = 1) regarded the illness starting point to be when family and friends first noticed symptoms

^b^Although classified as recovered, participants (*n* = 2) still experienced cognitive symptoms related to AN

### Labeling

The majority of participants (*n* = 10) reported specific labels that they liked (*n* = 7), felt indifferent to (*n* = 4) and disliked (*n* = 3) when referring to the prolonged form of the AN illness. The remaining participant (*n* = 1) chose not to comment on a label.

The most preferred label amongst participants (*n* = 6) was ‘severe and enduring’ with reasons for liking this label including it reflecting their experience (*n* = 3), being more appropriate (*n* = 1) or more helpful (*n* = 1) than other labels used, and the attention that has come from the use of the label (*n* = 1):

I only came across uhmm, ‘severe and enduring’ about a year ago, uhmm, I had never heard the term before and I was very excited to come across it cos I was like this is, I mean, none of the labels have ever fit me perfectly, uhmm but I was like I’m so glad people are thinking of this as a, as a thing rather than just a failure to get better. – Participant 5

Despite this preference, a couple of these same participants (*n* = 2) described the ‘enduring’ part of the label as more reflective of their personal experience than the term ‘severe’. Reasons for this included difficulty acknowledging the severity aspect of their condition (*n* = 1) or the changeability of severity that can occur over time (*n* = 1):

…I find the ‘severe’ part of it is the bit that I, that I feel least comfortable with…the physical severity is so changeable you know, week to week, month to month uhmm you know and I certainly don’t look the way I did when I was severely ill, yeah, but the ‘enduring’ part absolutely. – Participant 5

The use of the label ‘chronic’ caused division amongst participants (*n* = 8). Some of the participants reported to like (*n* = 1), feel indifferent towards (*n* = 4) or dislike (*n* = 3) this label for long-standing AN. The reason for preferring the term ‘chronic’ (*n* = 1) was the belief that it reflected the long-term trajectory and complexity of the illness:

When you first get it, it could be something that you can solve pretty much straight away but the longer you’ve got it the more intense and the more complex it is so maybe, yeah, that’s why I use the word ‘chronic’ cos its, its, I’ve had it for such a long time it’s much more than what it was initially. – Participant 4

The reason why participants (*n* = 3) reported disliking the label ‘chronic’ was due to the negative connotations associated with the term including the lack of hope it reflects in terms of the prospects of recovery:

I was being called a ‘career anorexic’ by a psychiatrist quite early on and then, like, people saying, quite often using that label ‘chronic’, its not like, there’s no hope in that… – Participant 3

Concern was also expressed by participants (*n* = 4) over the repercussions of using a label that segregates individuals from the overarching illness of AN. Reasons included a label communicating to health professionals that these patients have little prospects of recovering (*n* = 2), encouraging competition amongst patients (*n* = 1) and negatively impacting social status (*n* = 1):

For me, I’m probably a little bit resistant to some of the, the medicalised terminology and I just wonder about the term ‘severe and enduring’, whether it’s a little bit pathologising. – Participant 8

### Defining

There was a degree of ambivalence in some participants (*n* = 5) as to the appropriateness of providing universal criteria for this form of the illness. Reasons for uncertainty included the possibility of both positive and negative outcomes occurring as a result (*n* = 2) and scepticism as to the ability to apply consistent criteria across experiences (*n* = 3):

…I feel like it’s such a variable thing from person to person right uhmm and you know, if they, if they talk about it as an average then it is an average, although how you’d measure such things. – Participant 5

Despite ambivalence, these participants (*n* = 5) provided insights into what aspects of the illness they felt that health professionals should incorporate into a definition for long-standing AN if it were to occur. The other participants (*n* = 6) expressed either certainty for (*n* = 2) or against (*n* = 1) the appropriateness of defining this form of the illness, with the remaining participants (*n* = 3) choosing not to comment.

For participants (*n* = 8) providing an opinion on the defining characteristics of long-standing AN, the majority (*n* = 5) reported duration of illness to be an appropriate criterion. When providing a specific duration, it was reported that either a seven-year period (*n* = 3) or a ten or more year period with AN (*n* = 1) was most appropriate. For the remaining participant (*n* = 1), there was uncertainty regarding a specific time frame given the transition was described to be such an individual process:

There is probably a point, and I don’t know what that point is, and it’s probably different times for everyone, where it does stop being, you know, something that is acute that you know, one round of treatment or two rounds of treatment can treat and you recover, to where it actually becomes, ok, this is a serious problem. – Participant 11

Duration of illness was deemed by some participants (*n* = 3) as an inappropriate criterion to incorporate into a definition. Reasons against its inclusion was that each experience was entirely different making a specified illness duration futile (*n* = 1), concerns that young people may meet this criterion and be diagnosed (*n* = 1) and currently inadequate knowledge of the subgroup to provide a specific time frame (*n* = 1). One suggestion made (*n* = 1) to address the concern over younger patients meeting criterion that is illness duration-dependent is to provide a minimum age bracket for a diagnosis to be made.

Other criteria described to be appropriate to include in a definition was the presence of physical and irreversible health complications caused by AN (*n* = 2), the degree of entrenchment of the illness in the lives of patients (*n* = 1) and the number of unsuccessful treatment attempts (*n* = 1). The latter criterion was recommended to include not only length of treatment but also a measure of how “intractable” the illness was as well as the degree of patient engagement:

…it not only relates to length of treatment and all those kinds of things but also how intractable it seems to be or how difficult it is to treat or whether people engage or not, but I think it’s an important label and it is a distinction that needs to be made. – Participant 6

In contrast, however, a dislike for number of unsuccessful treatment attempts to be included was reported (*n* = 1) given the complexity that could come if multiple treatments were attempted within a short duration of time:

…I wouldn’t base it on how many treatment attempts because that can happen multiple times in a year. – Participant 3

Another criterion participants (*n* = 2) disliked were low body weight with the argument made that the physical manifestation of a disorder does not always reflect the complexity of the illness psychologically:

…a lot of people think, ‘Oh, someone’s a worse anorexic because they weigh less’ and that, and that’s just what other people can see, that’s that external manifestation without people realising what’s going on in your brain, you know, and severity’s not related necessarily to the weight. – Participant 11

## Discussion

The aim of the current study was to investigate the perspectives of individuals with a lived experience on the labeling and defining of long-standing AN. The preferences for representing the illness in the lives of the participants varied based on stories and the impact these experiences had when shaping their relationship to the long-standing eating disorder. Despite these variable experiences, similarities were identified in relation to preferences for both labeling and defining the illness, together with the impact of a classification system on other individuals who were experiencing this stage of AN. A common theme uncovered when investigating both questions were concerns regarding the negative repercussions that might emanate from segregating these individuals from the overarching AN disorder and the unattainability of a single definition being applied to these complex presentations. Despite these concerns, the current study reflects lived experience perspectives so that the individuals most affected by the illness can finally contribute to this debate.

The first question related to the labeling of the illness. Only two terms were reported by the lived experience sample when discussing their likes and dislikes for a label, which included ‘severe and enduring’ as the most popular label with mixed perspectives described for the use of the term ‘chronic’. When comparing this to the field of research, it was found that the term ‘chronic’ was most commonly applied to these individuals followed second by ‘severe and enduring’ [7]. The reason for disliking the term ‘chronic’ in the sample of lived experience individuals was reportedly the negative connotations that have come to be associated with its use, which has been a similar concern reported by researchers in the field [18, 37, 38]. Therefore, the current findings reflect the variation of individual perspectives, although there is a clear preference for the use of the label ‘severe and enduring’ for the later stage of the illness.

In relation to the second question of how to best define long-standing AN, there were similar findings in the current study to criteria that has been commonly applied by researchers in the field [12, 17, 18]. Illness duration was the most preferred criterion for individuals with a lived experience with many reporting a seven-year time frame as the minimum period symptoms should be experienced in order for it to be considered a long-standing disorder. This was also the most commonly applied time frame found in the literature for this particular subgroup [7]. With preliminary evidence suggesting shifts can occur as the illness transitions from earlier to later stages [24], research recruiting a large sample could help determine if the seven-year time frame is a useful indicator for identifying this form of the illness in a significant proportion of the long-standing AN population.

The suggestion to implement a minimum age bracket if illness duration were to be used as criterion is something that is worth further investigation when defining long-standing AN. In the diagnosis of other mental health conditions, such as Antisocial Personality Disorder, the DSM-5 requires an individual to be aged at least 18 years in order to meet diagnostic criteria [2]. Alternative classification systems, such as the separate category of Conduct Disorder, has been made available for younger individuals who demonstrate repetitive and persistent behaviour that is antisocial, aggressive and/or defiant in nature, in order to prevent younger individuals from being diagnosed with a personality disorder [39–41]. With similar concerns expressed for diagnosing young individuals with a later stage of AN, adopting an alternative classification system such as that which has been used for other mental health disorders, could be useful when defining stages of illness in the field of eating disorders. A minimum age bracket may exclude younger individuals from being classified as experiencing a long-standing illness and prevent them from receiving alternative treatment options aimed at the later stage of the disorder (e.g. [12, 42]).

Some criteria suggested by participants overlapped with findings by Tierney & Fox [11]. This included the criterion of previously failed treatment attempts, which was endorsed as important in patients by a sample of professional experts [11] and met with mixed opinions by participants in the current study. The suggestion to include the degree of entrenchment of AN by a participant in the current study may overlap with the criteria of entrenched ‘anorexic’ cognitions and an intertwined identity with the disorder, which were endorsed in the sample of practitioners recruited by Tierney and Fox [11]. A unique finding in the current study was the suggested criterion of irreversible health complications, which had not been reported as part of a definition in previous literature included in the systematic review by Broomfield et al. [7].

Findings from the current study that was in contrast to previous research included low body weight and the impact of psychological symptoms being potentially useful criteria in the identification of long-standing AN. There was a preference against using low body weight as criterion by participants in the current study, which was inconsistent with what was regarded as useful by practitioners [11] and adopted when defining this form of the illness in previous research [43–45]. When considering empirical evidence on body weight, a study by Raykos et al. [46] investigated the effect of pre-treatment BMI on quality of life outcomes when treating severe and enduring participants with enhanced cognitive-behaviour therapy [47]. In this particular study, it was found that BMI was a poor indicator for differentiating participants at earlier compared to later stages of the AN disorder [46]. Although further research is required, there is so far a lack of evidence to suggest that low body weight is a valid or preferred indicator for the later stage of the illness.

With empirical evidence suggesting that psychological symptoms may be useful indicators for severity of illness in long-standing AN [19, 20], participants in the current study did not describe a preference for these symptoms as part of a definition for the illness. However, a reason provided for disliking low body weight as criterion was its reflection of only physical manifestations of AN without considering the internal symptoms. Perhaps the reference to internal symptoms included psychological distress that has recently been explored empirically [19, 20]. Investigating the use of psychological indicators of distress needs to be further explored using a mixed methods approach given participants with lived experience may provide information on internal symptoms that cannot be identified through quantitative methods alone.

One of the more surprising findings from the current study was the identification of the term ‘enduring’ as being more relevant to the experience of some participants than the term ‘severe’. This finding invites the question of whether multiple subgroups exist within the later stage of AN as opposed to one single homogenous group. It was suggested by Wildes et al. [19] that some presentations of AN may be enduring but not severe and vice versa. With the current study utilising the inclusion criterion most commonly found in the literature, which is an illness duration of seven or more years [7], it is possible that this research only recruited individuals who had an enduring condition without necessarily having a severe disorder. A consensus as to what constitutes severity is required, with some suggesting that a long-term experience of AN qualifies as being severe [17, 48, 49] whilst others have suggested they are distinct constructs [19, 20, 46]. Additional research is essential for determining whether individuals at the later stage of the illness share characteristics that allow for a unified classification system or whether it is instead useful to further divide individuals with long-standing AN based on symptomatology.

Limitations of this research include recruitment through a convenience sampling method with the snowball technique potentially biasing responses. Additionally, recruitment for the current study involved advertising using the label ‘severe and enduring’ as well as the inclusion criterion of an illness duration of seven or more years. The title and inclusion criterion may have influenced the results as it could have biased participants in their answers and attracted more individuals with preference for this label and criterion to participate. Furthermore, there is a limit to the generalizability of these findings to other genders with only females participating in this research. A major strength of the current study was that thematic saturation was demonstrated during the coding process, which is when recruitment concluded. This was achieved after 11 participants were interviewed with previous qualitative research on this population recruiting a maximum of eight participants [18, 50, 51].

In terms of the practical implications of these findings, it is important that clinicians and researchers respect the individual preference of patients when referring to their illness given the variability found amongst participants in the current study. It is also critical that there is consideration into what inclusion criteria is being used when recruiting individuals with this form of the illness. Until it is confirmed whether long-standing AN is an homogenous group, researchers need to be mindful when selecting appropriate inclusion criteria and be explicit of the definition they apply for this population in all publications.

## Conclusion

It was the aim of the current study to provide insight into the perspectives of individuals with lived experience into the labeling and defining of the later stage of AN. Along with identifying a preference for the label ‘severe and enduring’ and the criterion of duration of illness, the study revealed concerns from these individuals with respect to the negative repercussions that may occur if this group were to become segregated from the overarching AN condition. Additionally, findings from the current study reveal the possibility that individuals in the later stage of the illness may not exist within an homogenous group and thus making their identification difficult if applying the same criteria. It is the goal of the authors that future research will use the perspectives of both practitioners and individuals with lived experience when determining appropriate labeling and defining features for long-standing AN.

## Data Availability

Due to privacy and ethical considerations relating to maintaining the confidentiality of participants, data cannot be made available.

## Abbreviations

AN: Anorexia Nervosa
DSM-5: Diagnostic and Statistical Manual of Mental Disorders, Fifth Edition (American Psychiatric Association, 2013)

## Footnotes

1 The term ‘long-standing AN’ has been adopted in the current study as a neutral reference to the later stage of the illness.
